# Correlation between the Injury Site and Trauma Mechanism in Severely Injured Patients with Blunt Trauma

**DOI:** 10.1155/2022/8372012

**Published:** 2022-05-24

**Authors:** Young Un Choi, Sung Woo Jang, Su Hyun Kim, Ji Wool Ko, Myoung Jun Kim, Hongjin Shim, Jae Hun Han, Ji Hye Lim, Kwangmin Kim

**Affiliations:** ^1^Department of Surgery, Yonsei University Wonju College of Medicine, Wonju 26426, Republic of Korea; ^2^Trauma Center, Wonju Severance Christian Hospital, Wonju 26426, Republic of Korea; ^3^Wonju Severance Trauma Research Group, Yonsei University Wonju College of Medicine, Wonju 26426, Republic of Korea; ^4^National Health Big Data Clinical Research Institute, Yonsei University Wonju College of Medicine, Wonju 26426, Republic of Korea

## Abstract

**Background:**

In patients with severe injury, predicting the injury site without using advanced diagnostic modalities can help formulate a diagnosis and treatment plan based on the suspected injury site.

**Objectives:**

This study aimed to determine the correlation between the injury site and trauma mechanism in severely injured patients with blunt trauma.

**Methods:**

We retrospectively analyzed the clinical characteristics—including age, sex, date of emergency room (ER) visit, time of injury, trauma mechanism (car accident, motorcycle accident, bicycle accident, pedestrian accident, fall, slipping and rolling down, crush injury, assault, and others), final diagnosis, injury severity score, abbreviated injury scale (AIS) score, and injury site—of 1,245 patients in a tertiary trauma center.

**Results:**

There was a strong correlation between certain injury sites and specific trauma mechanisms. In particular, most trauma mechanisms were associated with injury to the head and neck, as well as the chest, with a combined frequency of >40.0%. Moreover, when using one-way analysis of variance and Bonferroni's post hoc tests, there were significant differences in AIS scores 1, 3, 4, and 5 for each trauma mechanism.

**Conclusion:**

Generally, when patients with severe injury present to the ER, the injury site can be predicted upon initial assessment based on the trauma mechanism. Based on our study, the injury site predicted by a specific mechanism should be checked repeatedly and additionally through physical examination and imaging tools. This can reduce misdiagnosis and help with accurate diagnosis and treatment.

## 1. Introduction

Appropriate initial evaluation and subsequent management of severe injuries are crucial for obtaining favorable patient outcomes. Prompt recognition of physiological and anatomical anomalies helps initiate appropriate resuscitation and maintain the patient's vital signs. In particular, accurate diagnosis and the subsequent reduction in the time to definite care play important roles in preventing death and life-threatening morbidity [1]. However, when patients with severe injuries initially present to the hospital, life-threatening conditions that lead to unstable vital signs are frequently missed despite the presence of dedicated, well-trained trauma personnel who follow advanced trauma life support guidelines [2]. This results in the aggravation of a patient's condition. Furthermore, a definite diagnosis may not be established, even after physical examination, portable radiography, or focused assessment with sonography for trauma scans. Notably, the use of diagnostic modalities such as computerized tomography (CT) and angiography is limited in patients with unstable vital signs. To overcome this, a hybrid emergency room (ER) equipped with a CT machine and operating theater has been established and used for unstable patients [3]. However, many trauma centers in Korea operate without a hybrid ER. These factors delay the diagnosis and treatment of patients with unstable vital signs. By predicting the injury site in patients with severe injuries without using advanced diagnostic procedures, the treatment plan and diagnostic procedures could be determined based on the suspected injury site. This study aimed to determine the correlation between the injury site and the trauma mechanism in severely injured patients with blunt trauma.

## 2. Materials and Methods

The medical data of patients with blunt trauma were prospectively collected from the Korean Trauma Data Bank and retrospectively analyzed. We initially included 3,869 patients who visited the ER between January 1, 2018, and December 31, 2020. These patients were screened by the trauma team. The screening criteria are presented in Appendix 1. Patients with blunt trauma with an injury severity score (ISS) of ≥16 were included in the study. All patients who were dead on arrival, had experienced thermal injury, or had been injured via an unknown mechanism were excluded from the study. Finally, a total of 1,245 patients were included in the analysis. This retrospective study was approved by the institutional review board of the tertiary care hospital (IRB no. CR321156). Owing to the retrospective nature of the study and the use of anonymized data, the requirement for informed patient consent was waived.

We collected and analyzed data on the patients' clinical characteristics, such as age, sex, date of ER visit, time of injury, trauma mechanism (car accident, motorcycle accident, bicycle accident, pedestrian accident, fall, slipping and rolling down, crush injury, assault, and others), final diagnosis, ISS, abbreviated injury scale (AIS) score, and injury site. Based on age, the participants were grouped into adolescents (<18 years), adults (18–65 years), and older adults (>65 years). The AIS score was used to categorize patients according to their injury site (head and neck, face, chest, abdomen, extremities, and external).

### 2.1. Statistical Analysis

Categorical variables were expressed as frequencies and percentages. A one-way analysis of variance (ANOVA) test was used to analyze the differences in means between the groups. A Bonferroni post hoc test was performed to determine statistically significant differences between the groups. All statistical analyses were performed using SAS software version 9.4 (SAS, Cary, North Carolina, USA), and statistical significance was set at *P* < 0.05.

## 3. Results

**Figure 1:**
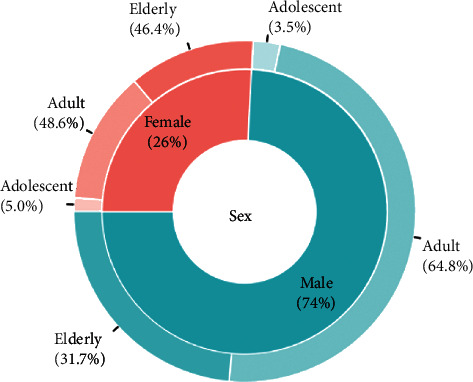
The patients' age and sex distribution.

**Figure 2:**
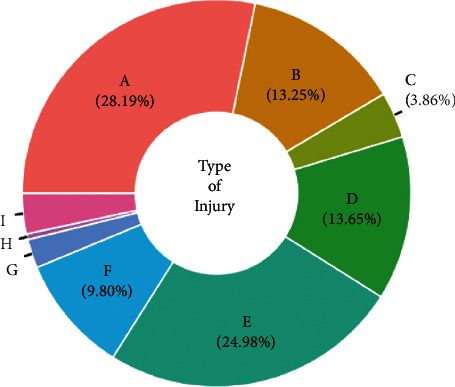
The percentage of patients that experienced each type of trauma mechanism (A, car accident; B, motorcycle accident; C, bicycle accident; D, pedestrian accident; E, fall; F, slipping and rolling down; G, crush injury; H, assault; I, others).

**Figure 3:**
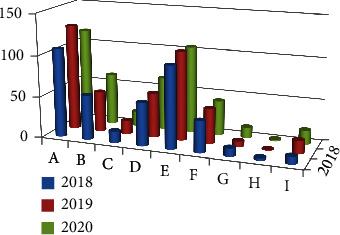
The number of patients injured by each trauma mechanism in 2018, 2019, and 2020. There was a similar trend in the distribution of patients by trauma mechanism between 2018 and 2020 (A, car accident; B, motorcycle accident; C, bicycle accident; D, pedestrian accident; E, fall; F, slipping and rolling down; G, crush injury; H, assault; I, others).

**Figure 4:**
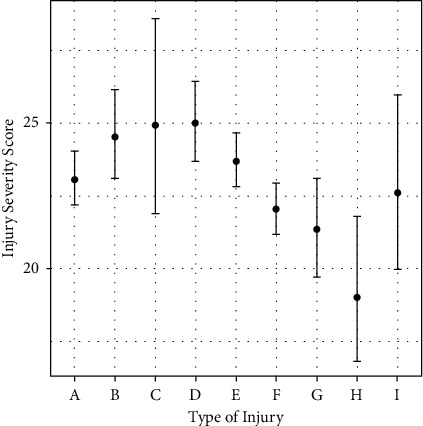
Mean (± standard deviation) injury severity score by trauma mechanism (A, car accident; B, motorcycle accident; C, bicycle accident; D, pedestrian accident; E, fall; F, slipping and rolling down; G, crush injury; H, assault; I, others).

Of the 1,245 patients, 924 (74.2%) were men. Among the men, 3.5% were adolescents, 64.8% were adults, and 31.7% were older adults (Figure 1). The most common trauma mechanisms were car accidents (28.19%), falls (24.98%), pedestrian accidents (13.65%), and motorcycle accidents (13.25%) (Figure 2). The numbers of patients injured via each trauma mechanism in 2018, 2019, and 2020 are shown in Figure 3. There was a similar trend in the distribution of patients by trauma mechanism across the three years. The highest and lowest mean ISS occurred due to pedestrian accidents (25 ± 9.3) and assaults (19.0 ± 3.4), respectively. The mean ISS according to the trauma mechanism is shown in Figure 4 and Table 1. There was a strong correlation between certain injury sites and specific trauma mechanisms. In particular, most trauma mechanisms were associated with injury to the head and neck, as well as the chest, with a combined frequency of >40.0% (Table 1). From Table 1, we can see that the frequency of specific injury sites is different for each trauma mechanism.

In addition, a one-way ANOVA was performed by comparing each injury site with different trauma mechanisms to determine the differences in the mean AIS scores for the four AIS groups with the highest frequencies in all trauma mechanisms (AIS scores 1, 3, 4, and 5) (Table 2). AIS scores 1, 3, 4, and 5 were found to be independently significant. This means that the severity of the injury site is different for each trauma mechanism. As a result, the AIS difference in frequency and severity indicates that the specific severe injury sites predicted for each trauma mechanism can be expected. Thereafter, a Bonferroni post hoc test was performed to check whether there was a difference in AIS between the traffic accident group and the non-traffic accident group.

There were significant differences in the mean AIS scores 1, 3, 4, and 5 between the traffic accident group, comprising trauma mechanisms A, B, C, and D, and the non-traffic accident/control group, comprising trauma mechanisms E, F, G, H, and I (Table 3). This shows that AIS scores 1, 3, 4, and 5 are significantly higher in the traffic accident group than in the non-traffic accident group. As seen in the above results, a specific injury site is marked as severe in the traffic accident group. All other details are presented in Table 1.

## 4. Discussion

In patients who experienced road traffic accidents, including car, motorcycle, bicycle, and pedestrian accidents, the most common injury sites were the head, neck, and chest. During impact, a car's steering wheel and airbags may collide with the driver's chest wall and the internal rear-view mirror with the driver's head, whereas motorcycle, bicycle, and pedestrian accidents may cause a person's upper body to hit the ground. Furthermore, a car may directly impact a pedestrian's extremities and the pelvic girdle. A previous study in Brazil reported that severe chest injuries were common among patients involved in car accidents, while head and extremity injuries were common among those involved in pedestrian accidents [4]. In recent years, studies have been conducted on the development of tools that simulate car accidents, such as the MADYMO (mathematical dynamic models) program. Various tools for predicting injury sites and types have subsequently been developed. However, further studies investigating such tools are necessary to accurately predict injury sites according to the accident type.

Traffic accidents are the most common cause of trauma in Korea, and the number of patients with severe injury per annum has remained unchanged. Notably, this trend has been observed not only in this study but also in other nationwide studies. National data show that although the total number of traffic accidents and patients with injury has not reduced, the mortality rate has declined [5]. This is likely owing to improvements in hospital transfer following traffic accidents and in-hospital management of patients with trauma. Although the number of pedestrian accident-related deaths has remained constant, the total number of deaths has decreased. This implies that the number of car accident-related deaths has decreased, possibly as a result of improvements in car safety systems and patient management after trauma. Consequently, a system for reducing pedestrian accident-related deaths is necessary. Lowering the speed limit may be an effective strategy. In developed countries such as the United States, when the speed limit has been gradually lowered, the incidence of traffic accidents and life-threatening injuries has decreased [6, 7]. Lowering the speed limit may reduce the frequency and severity of pedestrian and vehicular accidents. In most countries, the speed limit is 50 km/h on general roads in urban areas, except in Japan, where it is 60 km/h. In Sweden, after lowering the speed limit to 50 km/h in urban areas in 2008, the traffic accident-related mortality rate decreased [8]. This new limit became the standard speed limit throughout Europe. Consequently, a speed limit of 30 km/h was strictly enforced in England, the Netherlands, Germany, and France. In Illinois, the speed limit is 48 km/h in urban areas and 24 km/h in alleys [9]. Similarly, in California, the speed limit is 40 km/h in residential and business districts, school zones, and playground areas [10]. In addition, in Yeongdo-gu, Busan, there was a decrease in the number of traffic accident-related deaths when a reduced speed limit of 50 km/h was trialed in 2017 [11]. Since April 17, 2021, the speed limits have been lowered to 50 km/h in the downtown areas and 30 km/h in the residential areas of South Korea. This strategy is expected to gradually reduce the number of accidents.

In this study, workplace accidents were found to be a common cause of falls. In Korea, more men than women perform physical labor. Therefore, it was not surprising that such injuries occurred more commonly in men than in women (Figure 1). In addition, such accidents occurred more often during the day than at night, possibly because industrial accidents and falls from a ladder or roof are more common during work hours. Industrial accident statistics show that slipping is the most common trauma mechanism (20,101 patients, 21.37%), followed by falls (15,103 patients, 16.06%) [12]. In addition, these statistics showed that there were annual increases in the total number of patients who experienced industrial accidents. Here, the same pattern was noted, whereby 52, 24, and 64 patients had industrial accidents in 2018, 2019, and 2020, respectively. This highlights the need for adequate training on the proper use of industrial facilities and the importance of following safety rules.

Of the patients who had slipped, 83% had a head and neck injury with an AIS1 score of ≥3. As head injuries are usually severe, ER trauma physicians should consider the possibility of head injuries in patients who have slipped. Crush injuries commonly occurred during tree logging or from trauma to the head from heavy metal objects during construction. Here, we considered pelvic girdle injuries as extremity injuries. Pelvic injuries are common in patients with crush injuries, usually caused by machines. Although six patients presented with head and neck injuries after being assaulted, this was noted in only a small number of patients, and therefore, the implications of this finding are limited. Following their arrival at the ER, three of the six patients experienced long-term admission, despite being stable and alert. This suggests that their head and neck injuries were detected through imaging. This highlights the importance of using diagnostic imaging in patients with assault injuries.

This study had some limitations, including certain confounding factors that should be considered when interpreting our findings. For example, the injury site of a patient who experienced a pedestrian accident may depend on whether the vehicle was slowing down at the time of the accident. Similarly, among patients who had bicycle accidents, the injury site might depend on whether the cyclist was wearing a helmet at the time of the accident. In addition to trauma mechanisms, complex factors such as these should be considered. Nevertheless, the correlation between the injury site and the trauma mechanism should be evaluated to predict possible injury sites. A previous study determined the predictive value of trauma mechanisms and investigated the associations between trauma mechanisms and injury sites as well as the patients' treatment plans and outcomes, such as the need for surgery within 24 h, intensive care unit admission, and death within 24 h [13]. The commonality between the previous study and our study is that we confirmed the significance of trauma mechanisms.

We also investigated the monthly differences in the number of patients with injuries and the mean ISS; however, no monthly or seasonal differences were noted (data not shown). This differs from the findings of a previous study in Shiraz, Iran, where the number of patients with injury doubled in summer compared with that in winter [14].

The ISS represents injury severity, and a high ISS has been associated with a higher mortality rate, poorer clinical course, and increased length of hospital stay [15]. Here, we defined severe injury as an ISS of ≥15. The AIS score is based on the anatomical location of the injury. The body is divided into six regions, and the severity of an injury is divided into six degrees: minor, moderate, serious, severe, critical, and maximal. The ISS is calculated using the AIS score as follows: the highest AIS scores of the three most severely injured regions of the body are squared, and the three squared scores are summated (ISS = A^2^ + B^2^ + C^2^, where A, B, and C are the AIS scores of the three most injured regions of the body) [16]. A limitation of the ISS is its inability to reflect injury severity. Therefore, a new injury severity score (NISS) has been developed that is considered more accurate than the ISS for predicting patient outcomes [17]. Despite the NISS being easy to use, the ISS is still widely used.

As we only selected patients with severe injury (ISS ≥ 16) in this study, critically ill patients with trauma with a low ISS were excluded. For instance, patients with isolated grade III liver parenchymal injury, mesenteric rupture, and multiple bowel perforations were excluded because their ISS was 9. In contrast, patients with isolated bilateral hemopneumothorax were enrolled because their ISS was 16. However, patients with multiple bowel perforations may have a worse clinical course than those with bilateral hemopneumothorax. Therefore, a limitation of this study is that clinically severe patients may have been excluded owing to the inclusion criteria. Furthermore, among patients with external injury (AIS6), those with severe burns have an AIS6 score of ≥2. As we excluded patients with thermal injury, we could not analyze the correlation between trauma mechanisms and AIS6 scores. In fact, only one patient with an AIS6 score of 3 and two patients with an AIS6 score of 2 were enrolled in this study. Here, burns occurred in patients who experienced a car accident, explosion, or crush injury with friction caused by a machine.

## 5. Conclusions

In conclusion, specific trauma mechanisms in patients with severe blunt trauma were associated with a high frequency of injury at certain injury sites, especially the head, neck, and chest. Furthermore, the frequency of injury and AIS scores 1, 3, 4, and 5 were significantly high in all groups. In particular, the concentration of these injury sites was clearly shown in patients who suffered traffic accidents. Generally, when patients with severe injury are admitted to the ER, the severe injury site can be predicted upon initial assessment based on the trauma mechanism. Based on our study, the injury site predicted by a specific mechanism should be checked repeatedly and additionally through physical examination and imaging tools. This can reduce misdiagnosis and help with accurate diagnosis and treatment.

## Figures and Tables

**Table 1:** Patients' clinical characteristics analyzed by mechanisms.

	Overall^a^	A^a^	B^a^	C^a^	D^a^	E^a^	F^a^	G^a^	H^a^	I^a^
	(*N* = 1245)	(*N* = 351)	(*N* = 165)	(*N* = 48)	(*N* = 170)	(*N* = 311)	(*N* = 122)	(*N* = 30)	(*N* = 6)	(*N* = 42)
Age (years)	56.9 ± 18.3	54.3 ± 17.6	52.2 ± 22.7	62.0 ± 15.6	61.5 ± 18.9	56.3 ± 17.0	65.2 ± 15.8	54.9 ± 13.5	50.8 ± 14.6	55.5 ± 12.0
Sex										
Female	321 (25.8)	99 (28.2)	13 (7.9)	6 (12.5)	82 (48.2)	70 (22.5)	39 (32.0)	4 (13.3)	2 (33.3)	6 (14.3)
Male	924 (74.2)	252 (71.8)	152 (92.1)	42 (87.5)	88 (51.8)	241 (77.5)	83 (68.0)	26 (86.7)	4 (66.7)	36 (85.7)
ISS	23.6 ± 8.8	23.1 ± 8.7	24.5 ± 10.3	24.9 ± 12.5	25.0 ± 9.3	23.7 ± 8.2	22.1 ± 5.1	21.4 ± 4.9	19.0 ± 3.4	22.6 ± 10.2
AIS1	2.4 ± 2.0	1.9 ± 1.8	2.6 ± 2.0	3.1 ± 1.9	2.4 ± 2.0	2.5 ± 2.0	3.9 ± 1.6	2.1 ± 2.2	4.2 ± 0.4	0.5 ± 1.6
AIS1 ≥ 3	637 (51.2)	143 (40.7)	87 (52.7)	34 (70.8)	78 (45.9)	166 (53.4)	106 (86.9)	14 (46.7)	6 (100.0)	3 (7.1)
AIS2	0.6 ± 0.9	0.7 ± 0.9	0.8 ± 1.0	0.4 ± 0.7	0.7 ± 0.9	0.5 ± 1.0	0.2 ± 0.6	0.4 ± 0.7	0.5 ± 0.5	0.2 ± 0.5
AIS2 ≥ 3	32 (2.6)	12 (3.4)	9 (5.5)	0 (0.0)	5 (2.9)	6 (1.9)	0 (0.0)	0 (0.0)	0 (0.0)	0 (0.0)
AIS3	1.7 ± 1.7	2.1 ± 1.6	1.6 ± 1.5	1.6 ± 1.6	1.9 ± 1.7	1.9 ± 1.8	0.5 ± 1.4	1.6 ± 1.6	0.0 ± 0.0	2.0 ± 1.5
AIS3 ≥ 3	583 (46.8)	200 (57.0)	73 (44.2)	21 (43.8)	91 (53.5)	143 (46.0)	16 (13.1)	14 (46.7)	0 (0.0)	25 (59.5)
AIS4	1.1 ± 1.4	1.5 ± 1.6	0.9 ± 1.4	0.6 ± 1.3	1.0 ± 1.3	1.1 ± 1.4	0.3 ± 0.9	1.2 ± 1.7	0.3 ± 0.8	1.8 ± 1.5
AIS4 ≥ 3	255 (20.5)	106 (30.2)	28 (17.0)	6 (12.5)	27 (15.9)	56 (18.0)	7 (5.7)	9 (30.0)	0 (0.0)	16 (38.1)
AIS5	1.5 ± 1.5	1.5 ± 1.3	1.7 ± 1.4	0.9 ± 1.5	2.1 ± 1.7	1.5 ± 1.5	0.4 ± 1.0	0.8 ± 1.5	0.2 ± 0.4	2.6 ± 1.6
AIS5 ≥ 3	330 (26.5)	90 (25.6)	46 (27.9)	6 (12.5)	69 (40.6)	82 (26.4)	8 (6.6)	4 (13.3)	0 (0.0)	25 (59.5)
AIS6	0.0 ± 0.1	0.0 ± 0.2	0.0 ± 0.0	0.0 ± 0.0	0.0 ± 0.0	0.0 ± 0.0	0.0 ± 0.1	0.0 ± 0.0	0.0 ± 0.0	0.0 ± 0.3
AIS6 ≥ 3	1 (0.1)	1 (0.3)	0 (0.0)	0 (0.0)	0 (0.0)	0 (0.0)	0 (0.0)	0 (0.0)	0 (0.0)	0 (0.0)

A, car accident; B, motorcycle accident; C, bicycle accident; D, pedestrian accident; E, fall down; F, slip and rolling down; G, crush; H, assault; I, others; AIS, abbreviated injury scale; AIS1, head and neck AIS; AIS2, facial AIS; AIS3, chest AIS; AIS4, intra-abdominopelvic organ AIS; AIS5, extremity and pelvic bone AIS; AIS6, external AIS. ^a^Data are expressed as *n* (%) unless otherwise noted.

**Table 2:** The correlation between injury mechanisms and injury sites.

	AIS1	AIS3	AIS4	AIS5	*p* value
A	1.9 ± 1.8^a^	2.1 ± 1.6^b^	1.5 ± 1.6^a,b^	1.5 ± 1.3^a,b^	<0.001
B	2.6 ± 2.0^a^	1.6 ± 1.5^a,b^	0.9 ± 1.4^a,b,c^	1.7 ± 1.4^a,c^	<0.001
C	3.1 ± 1.9^a^	1.6 ± 1.6^a,b^	0.6 ± 1.3^a,b^	0.9 ± 1.5^a^	<0.001
D	2.4 ± 2.0^a^	1.9 ± 1.7^b^	1.0 ± 1.3^a,b,c^	2.1 ± 1.7^c^	<0.001
E	2.5 ± 2.0^a^	1.9 ± 1.8^a,b^	1.1 ± 1.4^a,b,c^	1.5 ± 1.5^a,b,c^	<0.001
F	3.9 ± 1.6^a^	0.5 ± 1.4^a^	0.3 ± 0.9^a^	0.4 ± 1.0^a^	<0.001
G	2.1 ± 2.2^a^	1.6 ± 1.6	1.2 ± 1.7	0.8 ± 1.5^a^	0.021
I	0.5 ± 1.6^a^	2.0 ± 1.5^a^	1.8 ± 1.5^a^	2.6 ± 1.6^a^	<0.001

A, car accident; B, motorcycle accident; C, bicycle accident; D, pedestrian accident; E, fall down; F, slip and rolling down; G, crush; H, assault; I, others; AIS, abbreviated injury scale; AIS1, head and neck AIS; AIS3, chest AIS; AIS4, intra-abdominopelvic organ AIS; AIS5, extremity and pelvic bone AIS. ^a^Significant difference was observed between AIS1 and AIS3 or AIS4 or AIS5 as a result of post hoc analysis. ^b^Significant difference was observed between AIS3 and AIS4 or AIS5 as a result of post hoc analysis. ^c^Significant difference was observed between AIS4 and AIS5 as a result of post hoc analysis.

**Table 3:** The correlation between traffic accident and injury sites.

	AIS1	AIS3	AIS4	AIS5	*p* value
Traffic accident	2.3 ± 1.9^a^	1.9 ± 1.6^a,b^	1.2 ± 1.5^a,b,c^	1.6 ± 1.5^a,b,c^	<0.001
Non-traffic accident	2.7 ± 2.1^a^	1.5 ± 1.8^a,b^	1.0 ± 1.4^a,b,c^	1.3 ± 1.5^a,c^	<0.001

AIS, abbreviated injury scale; AIS1, head and neck AIS; AIS3, chest AIS; AIS4, intra-abdominopelvic organ AIS; AIS5, extremity and pelvic bone AIS. ^a^Significant difference was observed between AIS1 and AIS3 or AIS4 or AIS5 as a result of post hoc analysis. ^b^Significant difference was observed between AIS3 and AIS4 or AIS5 as a result of post hoc analysis. ^c^Significant difference was observed between AIS4 and AIS5 as a result of post hoc analysis.

## Data Availability

The datasets used and/or analyzed during the current study are available from the corresponding author upon reasonable request.

## Supplementary Materials

Supplementary Materials Appendix 1: trauma team activation criteria.
