# Supplementary material for: Correlation between the Injury Site and Trauma Mechanism in Severely Injured Patients with Blunt Trauma
Source: Emerg Med Int. 2022 May 24;2022:8372012. doi: 10.1155/2022/8372012 (PMC10070019; doi:10.1155/2022/8372012)
Supplement: Supplementary Materials — Appendix 1: trauma team activation criteria. [file 8372012.f1.docx]

# APPENDIX 1

## Trauma team activation criteria

1. Physiologic criteria

A. Airway obstruction/respiratory failure

B. Intubated status before trauma center arrival

C. Adults: respiratory rate <10 breaths/min or >30 breaths/min

D. Adults: systolic blood pressure <90 mm Hg

E. Adults: heart rate >100 beats/min

F. Glasgow coma scale <13

2. Anatomical criteria

A. All penetrating injuries

I. Head and neck, chest, abdomen

II. Extremity: proximal to elbow or knee

B. Chest

I. Flail chest

C. Nervous system

I. Open or depressed skull fracture

II. Paralysis or suspected spinal cord injury

D. Extremity and pelvis

I. Pelvic bone fracture

II. Two or more proximal long bone fractures

III. Crushed, degloved, mangled, or pulseless extremity

IV. Amputation proximal to the wrist or ankle

3. Mechanism of injury

A. Automobile crash: death in the same passenger compartment

B. Automobile crash: ejection from automobile

C. Automobile crash >60 km/h

D. Automobile versus pedestrian injury >30 km/h

E. Time for evacuation of the victim from an automobile >20 min (Intrusion, including roof: >30

cm any site)

F. Motorcycle, bicycle crash >30 km/h

G. Fall

I. Adults: >6 m

II. Children: >3 m

H. Injury from an explosion

4. Attending trauma physician’s judgment

* The trauma team should be activated when the patient has more than one criterion.

* The trauma team activation is determined by the findings observed at the beginning of

the patient’s visit.
